# The challenge of developing human 3D organoids into medicines

**DOI:** 10.1186/s13287-020-1586-1

**Published:** 2020-03-04

**Authors:** Joaquim Vives, Laura Batlle-Morera

**Affiliations:** 1 Cell Therapy Service, Blood and Tissue Bank (BST), Barcelona, Catalonia Spain; 2 Musculoskeletal Tissue Engineering Group, Vall d’Hebron Research Institute (VHIR), Universitat Autònoma de Barcelona (UAB), Barcelona, Catalonia Spain; 3 Medicine Department, Universitat Autònoma de Barcelona (UAB), Barcelona, Catalonia Spain; 4 Core Facilities Program, Centre for Genomic Regulation (CRG), Barcelona, Catalonia Spain

**Keywords:** Good manufacturing practice, Organoid, Pancreas, Regenerative medicine, Tissue engineering, Regulatory and quality compliance

## Abstract

The capacity of organoids to generate complex 3D structures resembling organs is revolutionizing the fields of developmental and stem cell biology. We are currently establishing the foundations for translational applications of organoids such as drug screening, personalized medicine and launching the future of cell therapy using organoids. However, clinical translation of organoids into cell replacement therapies is halted due to (A) a few preclinical studies demonstrating their efficacy and (B) the lack of robust, reproducible, and scalable methods of production in compliance with current pharmaceutical standards. In this issue of *Stem Cell Research & Therapy* [ref], Dossena and collaborators present a validated bioprocess design for large-scale production of human pancreatic organoids from cadaveric tissue in accordance with current good manufacturing practice. The authors also propose a set of specifications of starting materials and critical quality attributes of final products that are of interest to other developments provided that this type of medicines are different than any other medicinal product due to their complex composition and living nature of the active ingredient. Although large-scale production of functional cells secreting insulin is still a challenge, the development of methods such as the one presented by Dossena and collaborators contributes to move toward clinical use of organoids in the treatment of type 1 diabetes and opens avenues for future clinical use of organoids in degenerative pathologies.

## Background

Organoids are three-dimensional (3D) cell structures grown in vitro from stem cells, primarily isolated either from biopsies or from pluripotent stem cells, that recapitulate key features of both the development and performance of native organs [1]. Cells forming organoids are able to differentiate, undergo lineage commitment, and acquire the specific tissue patterning to develop into several endoderm, mesoderm, and ectoderm-derived tissues, mimicking somehow their in vivo counterparts. The resulting structures are complex and provide a unique opportunity to model human organ development and disease in a system remarkably similar to that occurring in vivo.

Despite the extensive use of organoids in basic research, their translational biomedical application is currently restricted to drug testing [2, 3] and initial cell replacement strategies [4, 5]. However, the regulatory framework does not define specific guides or rules to such type of entities and, for instance, drug testing using organoids in Europe needs be done in accordance with a very generic “Guideline on the principles of regulatory acceptance of 3Rs (replacement, reduction, refinement) testing approaches” (EMA/CHMP/CVMP/JEG-3Rs/450091/2012) by the European Medicines Agency (EMA). Currently, the clinical use of organoids would need to meet the requirements for cell and gene-based therapies and produced the following good manufacturing practices (GMP) as any other pharmaceutical drug [6].

In any case, organoids hold a huge potential in regenerative medicine purposes if current limitations are overcome, namely, (A) clonal variation, developmental stage, pluri-/multi-potency, and chromosomal stability of stem cells; (B) reproducibility, accuracy, and scalability of the methodologies proposed; (C) meaningful functional assessment of resulting organoids; and (D) preclinical validation [7]. Only if these constraints are conveniently addressed, organoid technology will be readily translated into novel approaches for the replacement of severely damaged organs, such as the pancreas in patients with type 1 diabetes for instance. Indeed, the generation of human pancreas organoids (hPOs) in compliance with pharmaceutical standards is a challenge in view that currents methods are laborious, time-consuming, expensive and, as stated previously, lack reproducibility [8]. In this context, improvements in bioprocess design and definition of strategies in compliance with quality and regulatory guidelines are needed, being crucial the use of chemically defined serum-free culture media and the establishment of robust specifications of starting materials and the critical quality attributes (CQA) of final product [9, 10]. This can only be understood considering hPOs as a cell-based medicinal product, from a pharmaceutical production perspective, in compliance with current GMP guidelines.

### Quality compliance in the production of organoids

GMP in cell therapy refers to a system for ensuring that medicinal products are consistently manufactured and controlled according to quality standards, thus minimizing the risks involved in the production process that cannot be eliminated otherwise through testing the final drug product, so they are safe to be used in patients. To achieve this, GMP covers all aspects of production (e.g., starting materials, facilities, and equipment), personnel (e.g., training, hygiene of staff), and documentation (e.g., standard operating procedures and registers to prove that they are consistently followed at each step in the manufacturing process) [6]. In fact, protocols for the development of human-induced pluripotent stem cell (iPSC)-derived retinal organoids have been successfully adapted to GMP standards required for the generation of transplantable photoreceptor cells for future clinical applications [11]. Similarly, Takebe and collaborators have further developed their initial liver buds protocol for massive production and reproducibility making it ready for further GMP adaptation in order to scale up production in GMP facilities for transplantation purposes [12]. Moroever, the production of cells for human application greatly benefits from the use of fully defined, xeno-free conditions rather than media supplemented with sera, thus improving reproducibility and preventing xeno-mediated infection or immune rejection when transplanted into patients.

The generation of hPOs from the human pancreas is feasible, although not at the scale necessary to result in sufficient number of cells for clinical use [13]. In their recent paper, Dossena and collaborators fixed a series of in-process controls and improved media formulation by replacing R-spondin-1 (RSPO1) conditioned medium (CM) with recombinant RSPO1 protein, resulting in a GMP-compliant protocol intended to deliver a cryopreserved hPO for further use in allogeneic therapies for type 1 diabetes [14].

Similar to other cell-based therapies, Dossena and collaborators paid special attention to define the criteria of acceptance of the starting materials in order to minimize the risk of transmittable viral diseases and to ensure the procurement of healthy pancreatic tissue for the production of hPO (Table 1). To do so, pancreas biopsies are processed within 36 h from extraction from donors aged 18–65 years, beating heart and declared brain death, known cause of death, negative for HIV, HBV, HCV, anti-HBc, VDRL-TPHA, absence of risk factors, systemic infections, and diabetes, amylase levels within three times the normal range [14]. Similar to the original small-scale research method, the GMP-validated production process resulted in hPOs with equivalent dimensions, growth kinetics, metabolism, and cellular composition. In the characterization of hPOs, the authors reported the presence of ductal cells and ductal progenitors but differentiation into endocrine cells producing insulin was not demonstrated in this work. Remarkably, viability rates were always above 75% after thawing thus demonstrating the feasibility of generating a bank of cryopreserved organoids, which could be further differentiated, either in vitro or in vivo, into insulin-producing cells, as already shown to be feasible by other authors [15]. This is a key point that will need to be addressed next to ensure clinical success, provided that the potency of hPO generated in this work will rely on their capacity to generate insulin and restore normoglycemia.

**Table 1:** Quality product profile for hPO. Critical quality attributes of hPO and acceptance proposed by Dossena and collaborators that can be adapted in other developments of organoid production in accordance with pharmaceutical quality standards. Additional controls may include determination of endotoxins, mycoplasma, and sterility test according to the pharmacopeia. *Ab* antibody, *Ag* antigen, *HBc* hepatitis B core, *HBV* hepatitis B virus, *HCV* hepatitis C virus, *HIV* human immunodeficiency virus, *VDRL-TPHA* venereal disease research laboratory test-treponema pallidum hemagglutination test

In process controls	Acceptance criteria
Donor	
Serology (HIV, HBV, HCV, anti-HBc, VDRL-TPHA)	Negative
Risk factors, systemic infections, diabetes	Absence
Amylase levels	Within 3 times the normal range
Biopsy	
Transport	21 ± 4 °C
hPO	
Isolation	Within 36 h from biopsy
Identity (using ductal, mesenchymal, hematopoietic and endothelial markers)	Presence of ductal progenitors (PDX1^+^/SOX9^+^)
Metabolism	Normal glucose consumption and lactate production
Viability	≥ 70%
Potency	Capacity to differentiate into insulin-secreting cells
Sterility (BacTAlerT)	Sterile
Karyotype	Normal

### Outlook

Although further research is required in order to evaluate the therapeutic benefit of organoids in the context of diabetes or other pathologies, only the adherence to strict quality standards will ensure reproducibility. The efficacy and safety of organoid-based therapies will require appropriate cellular composition, proper engraftment and vascularization into the host and the demonstration of functional activity. Moreover, translation of organoid technologies into the clinics may benefit from the emergence of human leukocyte antigen (HLA)-homozygous iPSC initiatives, which hold the potential to foster the development of patient compatible regenerative therapeutic approaches that can embrace large-scale production of organoids and organoids biobanking [16]. Furthermore, the blending of organoid technology with 3D bioprinting and vascularization approaches may lead to macrostructures with the desired cellular composition for successful transplantation [17]. The combination of all these technologies is key for realizing the full potential of organoids as therapies.

## Data Availability

Not applicable.

## Abbreviations

3D: Three-dimensional
CM: Conditioned media.
CQA: Critical quality attributes
CRISPR: Clustered regularly interspaced short palindromic repeats
EMA: European Medicines Agency
GMP: Good manufacturing practice
HLA: Human leukocyte antigen
hPO: Human pancreas organoid
iPSC: Induced pluripotent stem cell
